# Prevalence and risk factors for non-alcoholic fatty liver in children and youth with obesity

**DOI:** 10.1186/s12887-017-0867-z

**Published:** 2017-04-26

**Authors:** Carolina Jimenez-Rivera, Stasia Hadjiyannakis, Jorge Davila, Julie Hurteau, Mary Aglipay, Nick Barrowman, Kristi B. Adamo

**Affiliations:** 1 Division of Gastroenterology, Hepatology and Nutrition, University of Ottawa, Ottawa, Canada; 2 Division of Endocrinology and Metabolism, Faculty of Health Sciences, University of Ottawa, Ottawa, Canada; 3 Diagnostic Imaging, University of Ottawa, Ottawa, Canada; 4 Research Institute, University of Ottawa, Ottawa, Canada; 5 Children’s Hospital of Eastern Ontario, Faculty of Health Sciences, University of Ottawa, Ottawa, Canada; 6 School of Human Kinetics, Faculty of Health Sciences, University of Ottawa, Ottawa, Canada

**Keywords:** Non-alcoholic fatty liver, Obesity, Dyslipidemia

## Abstract

**Background:**

Non- Alcoholic Fatty Liver (NAFL) is a spectrum of liver diseases (LD) that ranges from benign fatty infiltration of the liver to cirrhosis and hepatic failure. Hepatic ultrasound (US) and serum alanine aminotransferase (ALT) are often used as markers of NAFL. Our aim is to describe prevalence of NAFL and associated findings on ultrasound (US) and biochemical parameters in a population of children and adolescents with obesity at the Children’s Hospital of Eastern Ontario.

**Methods:**

Children with Obesity (BMI >95th percentile) ages 8–17 years presenting to the Endocrinology and Gastroenterology clinics, without underlying LD were prospectively recruited from 2009 to 2012. Fasting lipid profile, HOMA IR) and serum adiponectin levels were measured. NAFL was defined as ALT > 25 and >22 IU/mL (males and females respectively) and/or evidence of fatty infiltration by US. Logistic regression was performed to assess associations.

**Results:**

97 children with obesity included in the study (Male 43%). Mean age was 12.9 ± 3.2 years (84% were older than 10 y). Mean BMI-Z score was 3.8 ± 1.4. NAFL was identified in 85%(82/97) of participants. ALT was elevated in 61% of patients. Median triglyceride (TG) level was higher in children with NAFL(1.5 ± 0.9 vs. 1.1 ± 0.5 mmol/L, *p* = 0.01). Total cholesterol, HDL, LDL and Non HDL cholesterol were similar in both groups(*p* = 0.63, *p* = 0.98, *p* = 0.72 and *p* = 0.37 respectively). HOMA IR was ≥3.16 in 53% of children(55% in those with NAFL and 40% in those without NAFL). Median serum adiponectin was 11.2 μg/ml(IQR 7.3–18.3) in children with NAFL vs. 16.1 μg/ml(IQR 9.0–21.9) in those without NAFL(*p* = 0.23). Liver US was reported as normal in 30%, mild fatty infiltration in 38%, moderate in 20% and severe in 12%. TG were significantly higher(1.5 mmol/L vs. 1.0 mmol/L, *p* < 0.01) and HDL-C was lower(1.0 mmol/L vs. 1.1 mmol/L, *p* = 0.05) in children with moderate and severe NAFL by US. BMI-Z score, HOMA IR, serum adiponectin and HDL levels were not associated with NAFL, however TG were significantly associated(OR = 3.22 (95% CI: 1.01–10.25, *p* = 0.04)).

**Conclusion:**

NAFL is highly prevalent in obese children and youth. Elevated TG levels are associated with NAFL; these findings may serve as a noninvasive screening tool to help clinicians identify children with obesity needing liver biopsy and/or more aggressive therapeutic interventions.

## Background

Childhood obesity is one of the primary predictors of obesity in adults; more than two thirds of children with obesity will become obese adults [1–3]. Non-alcoholic fatty liver (NAFL), metabolic syndrome, type 2 diabetes mellitus, obstructive sleep apnea and cardiovascular disease are not only well-described complications of obesity in adults, but are also becoming increasingly recognized conditions in children.

NAFL disease is a clinical pathological condition characterized by broad spectrum of liver damage ranging from isolated steatosis to steatohepatitis and cirrhosis [4]. The prevalence of NAFL in obese children is not well known. A very wide range of prevalence has been reported in the literature. Ten to 77% of children with obesity have evidence of hepatic fatty infiltration by ultrasound and 25% of them have elevation of liver enzymes [5, 6]. Children with NAFL are usually asymptomatic, they may present with vague abdominal pain and incidental elevation of alanine aminotransferase (ALT) screened due to child’s body habitus [7, 8]. NAFL is uncommon in children under 8 years of age, the average age at presentation is 12 years [9]. Liver biopsy is the gold standard for diagnosing NAFL, nonetheless performing a liver biopsy is invasive and carries risks of complications and often requires patient’s admission post procedure [10, 11]. The aim of this study is to identify the prevalence of fatty liver by non-invasive methods in a pediatric population with obesity as well as evaluate risk factors for NAFL by associations with ultrasound (US) findings and biochemical parameters.

## Methods

Children 8–17 years old with obesity (Body Mass Index, BMI > 95th percentile for age and sex) were prospectively recruited from September 2009 and December 2012. The study was conducted at the Children’s Hospital of Eastern Ontario, a tertiary care academic center, affiliated with the University of Ottawa. Institutional Research Board approval was obtained to conduct this study. Written informed consent was obtained from parent/legal guardian of children under 16 years of age; children 16 years and above provided their own consent.

Body weight was measured using a balance beam scale, calibrated to the nearest 0.1 kg daily. Height was measured using a SECA stadiometer. BMI was calculated according to the following formula: BMI = weight in kg/height in metres [2]. A BMI z score was calculated using https://www.cdc.gov/growthcharts/ norms.

A baseline blood sample was obtained for fasting glucose, insulin, lipid profile including total cholesterol, low-density lipoprotein cholesterol (LDLC), triglycerides (TG), high-density lipoprotein cholesterol (HDLC), alanine aminotransferase and aspartate aminotransferase (ALT and AST respectively) and serum adiponectin. ALT >25 IU/L was considered abnormal in boys and >22 IU/L in girls [12]. Other causes of liver diseases were excluded in children with elevated liver enzymes by screening for hepatotropic viruses (hepatitis A, B and C, cytomegalovirus and Epstein Barr virus), alpha 1 antitrypsin (A1AT) levels as well as autoantibodies and immunoglobulin G to rule out A1AT deficiency and autoimmune hepatitis respectively. Patients were not included in the study if there was a history of alcohol intake or had any other type of liver diseases such as drug induced fatty liver and genetic disorders.

Insulin resistance was estimated using the Homeostasis Model Assessment (HOMA) formula, which calculates insulin resistance based on simultaneous fasting blood glucose and insulin levels [13]. The HOMA formula is calculated as fasting insulin (mU/ml) multiplied by fasting plasma glucose (mmol/L) divided by 22.5. Lower HOMA values indicate higher insulin sensitivity. The estimate obtained with HOMA correlates well (*r* = −0.91, *p* < 0.001) with measures of insulin resistance obtained from obese and non-obese children and adolescents with the use of the euglycemic-hyperinsulinemic clamp technique [14]. HOMA >3.16 was considered a marker of insulin resistance as described by Keskin, et al. [15]. Serum high molecular weight adiponectin was measured in duplicate with the MILLIPLEX MAP Panel A kit (Millipore, Billerica, MA) using Luminex xMAP technology as described by the manufacturer. Under our experimental conditions, the intra- and inter-assay CV were both <10%.

Hepatic fatty infiltration: Abdominal US determined the brightness of the liver echogenicity compared with the kidney, portal vein wall and diaphragm [16], to assess the degree and severity of fatty liver. All US were performed by one of the two radiologists involved in the study and reviewed separately by both radiologists (JD and JH). The radiologists were blinded to the blood test-results and clinical history of patients. In the event of discrepancy they discussed the findings and came to an agreement. A General Electric Logiq 9 ® machine was used in all cases (GE Healthcare, Quebec, Canada). Axial and sagittal images through the liver were acquired using a 5 MHz probe. A sagittal image in the mid-axillary plane including the liver and right kidney was obtained to assess the echogenicity of the liver in comparison to the kidney. Axial right paramedian images were used to assess the degree of echo penetration through the liver, the clarity of portal vessels and visualisation or the diaphragm. Depths, focus point, 2D gain, were kept constant for all studies as starting parameters. If the deep portions and vessels of the liver were not seen, then imaging parameters were adjusted and the images were repeated. Standard ultrasound scoring system identified children without signs of liver steatosis and those with different degrees of steatosis including mild, moderate and severe fatty infiltration.

Children were excluded if they had known underlying liver disease, and/or had an insurmountable language barrier that would prevent consent to participation.

NAFL was defined as a mild, moderate or severe fatty infiltration as demonstrated by liver US or ALT levels of >25 IU/L in males and >22 IU/L in females. Descriptive statistics were calculated for all patient characteristics. Univariate associations between NAFL and each potential risk factors including TG, HDLC, non HDLC, HOMA IR, total cholesterol, LCLC, BMI z score and serum adiponectin, were assessed using Student t-tests, Mann-Whitney U tests, or Pearson chi-squared tests where appropriate. Univariate logistic regression was also used to determined estimates of effect and respective confidence intervals for each covariate. Variables were log-transformed as appropriate in the univariate logistic regression. To evaluate the sensitivity and specificity of different thresholds of TG as a potential screening tool for NAFL, an ROC curve was constructed. The area under the ROC curve was computed, together with a 95% confidence interval. All analyses were completed using SPSS version 23 and R version 3.0.2,

## Results

A total of 98 children consented to participate and 97 were included in the study period, one patient was excluded due to incomplete biochemical test results. Of those, 42 (43%) were males. Mean age was 12.9 ± 3.2 years. Mean BMI-Z score was 3.8 ± 1.4. Median ALT was 26.0 IU/L (IQR 17.0–33.5); abnormal ALT was found in 59/98 (60.8%) children. Median AST was 26.5 IU/L (21.0–30.0). Investigations for underlying liver disease were negative. Patient characteristics are shown in Table 1.

**Table 1:** Patient characteristics

	*N*	
Age, mean (SD)	97	12.9 (3.2)
Age- Pubertal (10+ years), *n* (%)	97	81 (83.5)
Male, *n* (%)	97	42 (43.3)
Race, *n* (%)		
White	80	66 (82.5)
Black	80	6 (7.5)
Arab	80	6 (7.2)
Aboriginal	80	4 (5)
South Asia	80	3 (3.8)
BMI z score, mean (SD)	97	3.8 (1.4)
Comorbidities, *n*(%)		
Type 2 diabetes	84	0(0.0)
NAFLD	84	49 (58.3)
Sleep apnea	83	14 (16.9)
Pre-existing acanthosis nigricans	79	44 (55.7)
Blood levels		
Total Cholesterol (mmol/L), mean (SD)	97	4.4 (0.8)
Triglycerides (mmol/L), median (IQR)	96	1.3 (0.8–1.9)
HDLC (mmol/L), median (IQR)	97	1.0 (0.9–1.2)
LDLC, mean (SD)	96	2.6 (0.7)
Non HDL Cholesterol (mmol/L), mean (SD)	84	3.3 (0.8)
HOMAIR +3.16, *n*(%)	95	50 (52.6)
ALT (IU/mL), median (IQR)	97	26.0 (17.0–33.5)
AST(IU/mL), median (IQR)	95	26.0 (21.0–30.0)
Adiponectin, median (IQR)	96	11.3 (7.5–19.3)
NAFLD grading by US, *n*(%)	97	
Normal		29 (29.9)
Mild		37 (38.1)
Moderate		19 (19.6)
Severe		12 (12.4)

*N* number of patients with available data, *BMI* Body Mass Index, *NAFLD* Non-Alcoholic Fatty Liver Disease, *HDLC* High Density Lipoprotein Cholesterol, *LDLC* Low Density Lipoprotein Cholesterol, *HOMA IR* Homeostatic Model Assessment Insulin Resistance, *AST* Aspartate aminotransferase, *ALT* Alanine aminotransferase

There were 9 patients (out of 83 with available data) receiving medications, 2 were on oral corticosteroids, one of which was also receiving metformin, one on concomitant lowering lipid agent and metformin, one on both insulin and metformin, one on inulin alone and 4 on metformin alone.

**Fig. 1:**
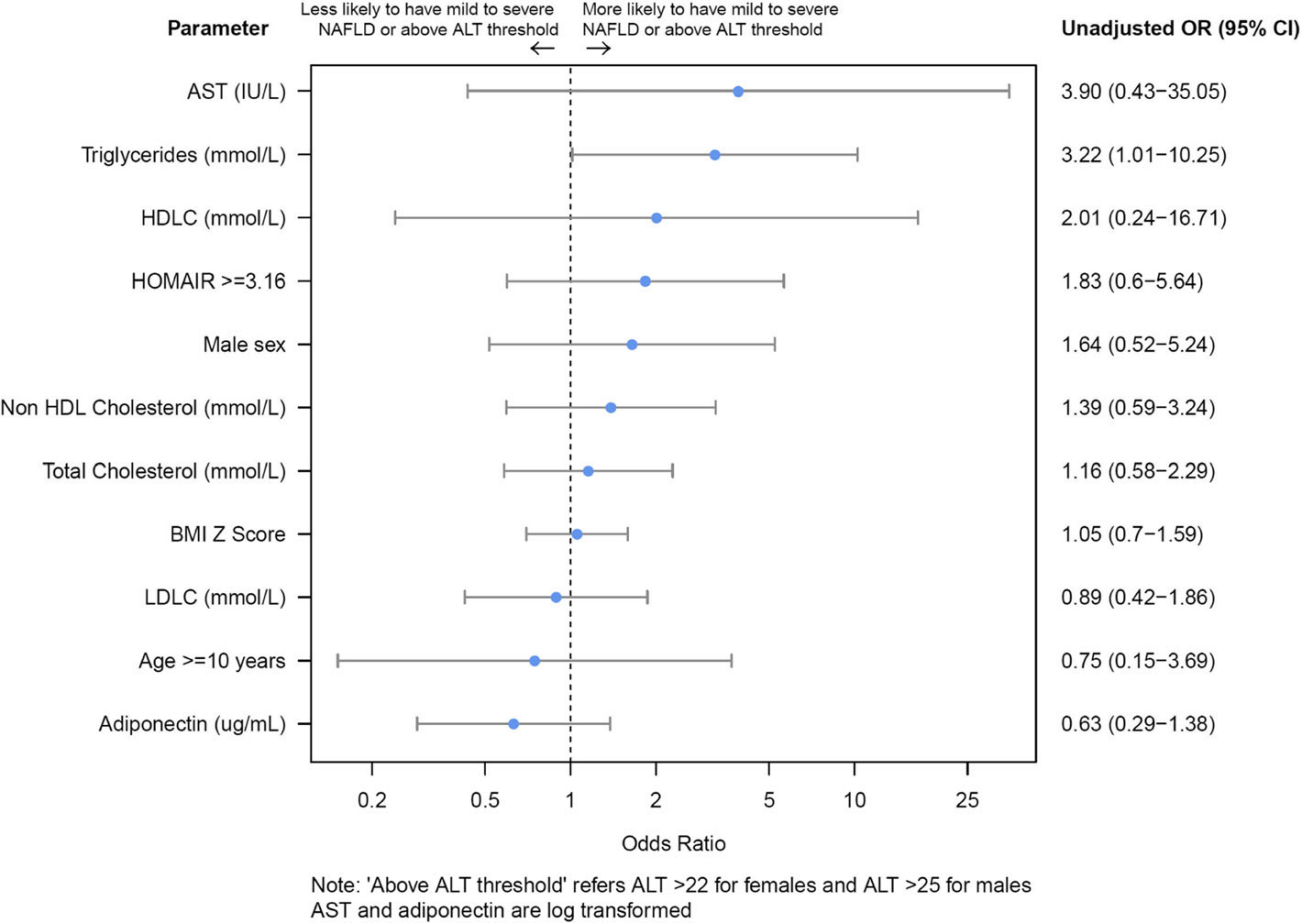
Forest graph showing Odds ratio for ALL patients with NAFL. *NAFL* Non-Alcoholic Fatty Liver, *AST* aspartate aminotransferase, *HDLC* High Density Lipoprotein Cholesterol, *HOMA IR* Homeostatic Model Assessment Insulin Resistance, *LDLC* Low Density lipoprotein Cholesterol, *BMI* Body Mass Index

Median TG level was 1.3 mmol/L (IQR 0.8–1.9), median HDL-C was 1.0 mmol/L (IQR 0.9–1.2), median LDLC was 2.50 mmol/L (2.1–3.1) median HOMA IR was 3.3 (IQR 2.0–5.8) and median serum adiponectin was 11.3 μg/ml (IQR 7.5–19.2). Elevated triglycerides were associated with NAFL in simple logistic regression (OR = 3.22, 95% CI = 1.01–10.25, *p* = 0.01). BMIZ score, HOMA IR, serum adiponectin and HDL levels were not associated with NAFL. Figure 1 shows odds ratios and associated 95% confidence intervals. There were no statistically significant associations between NAFL and AST, HDLC, HOMAIR, sex, non HDL cholesterol, total cholesterol, BMI Z score, LDLC, age or adiponectin. However, there was a significant association between TG and NAFL (OR = 3.22 (95% CI: 1.01–10.25), *p* = 0.04). To evaluate the sensitivity and specificity of TG as a potential screening tool for NAFL, an ROC curve was constructed. The area under the ROC curve was 0.69 (95% CI 0.54 to 0.84). An example screening cut off of TG > =0.755 gives a sensitivity of 90% and a specificity of 40% for predicting NAFL.

**Fig. 2:**
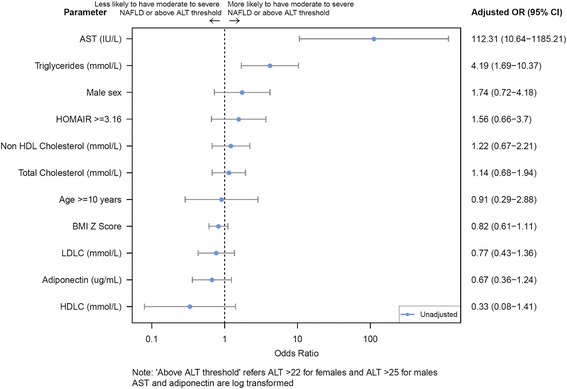
Forest graph for moderate to severe NAFL. *NAFL* Non-Alcoholic Fatty Liver, *AST* aspartate aminotransferase, *HDLC* High Density Lipoprotein Cholesterol, *HOMA IR* Homeostatic Model Assessment Insulin Resistance, *LDLC* Low Density lipoprotein Cholesterol, *BMI* Body Mass Index

Additional analysis was performed to assess whether risk factors could be subsequently identified when US detected moderate and severe fatty infiltration in combination with an abnormal ALT (Fig. 2). Once again TG were significantly higher in children with NAFL (1.5 mmol/L vs. 1.0 mmol/L, *p* < 0.01).

Liver US showed some degree of steatosis in 70% (69/98) of the cohort, varying from mild fatty infiltration in 38% (37/97), moderate in 20% (19/97) and severe 12% (12/97). Liver US was reported as normal in 30% (29/97) of children. Fourteen children with normal US had abnormal ALT (14/29, 48.3%). Two thirds of children with some degree of fatty infiltration by US had elevated ALT (45/68, 66.2%). Prevalence of NAFL (elevated ALT and US showing fatty infiltration) was 85% (82/97). Table 2 shows univariate associations between NAFL and selected risk factors.

**Table 2:** Univariate Associations between NAFLD definition and selected covariates

	*N*	Meets NAFLD definition: Any (mild/moderate/severe) fatty infiltration or ALT > 25 IU/mL (males)or ALT >22 IU/mL (females)		*P* Value
		No	Yes	
Sex, *n* (%)				0.57
Male	42	5 (11.9)	37 (88.1)	
Female	55	10 (18.2)	45 (81.8)	
Age (category), *n* (%)				1.00
< 10 Years	16	2 (12.5)	14 (87.5)	
> =10 Years	81	13 (16.0)	68 (84.0)	
Total Cholesterol (mmol/L), mean (SD) normal	97	4.3 (0.6)	4.4 (0.8)	0.63
Triglycerides (mmol/L), mean (SD)	96	1.1 (0.5)	1.5 (0.9)	0.01
HDLC (mmol/L), median (IQR)	97	1.0 (1.0–1.3)	1.0 (0.9–1.2)	0.98
LDLC (mmol/L), mean (SD) normal	96	2.7 (0.6)	2.6 (0.8)	0.72
Non HDL Cholesterol (mmol/L), mean (SD)	84	3.1 (0.6)	3.3 (0.8)	0.37
HOMAIR, *n* (%)	95			0.40
< 3.16		9 (20)	36 (80)	
> = 3.16		6 (12)	44 (88)	
AST(IU/mL), median (IQR)	95	23.5 (21.0–26.3)	27.0 (21.0–31.5)	0.18
Adiponectin, median (IQR)	96	16.1 (9.0–21.9)	11.2 (7.3–18.3)	0.23
BMI Z Score, mean (SD)	97	3.7 (1.3)	3.8 (1.4)	0.80

*NAFLD* Non-Alcoholic Fatty Liver Disease, *ALT* Alanine Aminotransferase, *HDLC* High Density Lipoprotein Cholesterol, *LDLC* Low Density Lipoprotein Cholesterol, *HOMA IR* Homeostatic Model Assessment Insulin Resistance, *AST* Aspartate aminotransferase, *BMI* Body Mass Index.

## Discussion

Childhood obesity is the main risk factor for the development of NAFL. Suspicion of NAFL is often based on patient’s body habitus, which may prompt clinicians to investigate further by assessing hepatic steatosis by liver US and measurement of ALT. Liver biopsy is the ideal test to establish the diagnosis [17], however this is invasive and access may be limited.

The prevalence of NAFL in pediatric patients with obesity by the definition used in this study was high (85%). Our finding was very similar to Xanthakos, et al. [18], where the prevalence of NAFL was 83% based on liver biopsies taken at the time of bariatric surgery. Another study [19] reported lower prevalence calculated between 20 and 30% when abnormal transaminases and ultrasound were used respectively, this may be related to a different definition of abnormal transaminases.

In the logistic regression analysis, it appeared TG levels were associated to NAFL. Given the broad confidence interval found, the association may or may not have clinical impact and it should be taken with caution. These findings were also demonstrated in a similar study by Navarro-Jarabo, et al. [20], where a higher level of triglycerides and higher BMI were related to hepatic steatosis. In addition to this, we looked more closely at children with moderate to severe steatosis by US and we found that HDLC was lower in this group of patients, which can be related to insulin resistance [21]. A recent study by Lee et al. [22], described the association between NAFL detected by US and hypertriglyceridemia among other biochemical and anthropometric parameters such as weight to height ratio and waist circumference amongst others.

Adiponectin, an adipocyte secreted protein, has been recognized for its physiological role in decreasing postprandial rise of free fatty acids and enhancement of insulin signalling (inhibits lipolysis in adipose tissue, stimulated free fatty acid oxidation in hepatocytes, stimulates insulin action in hepatocytes, inhibits gluconeogenesis, and improves insulin mediated glucose transport in skeletal muscle) [23]. Although secreted by the adipocyte, paradoxically adiponectin is reduced in obese adults [24], increased in anorexia nervosa and during periods of weight loss [25, 26]. There is a growing body of literature related to its role in the development of NAFL [23]. Since low adiponectin level was an indicator of NAFL in a large population of adults [27], we measured levels of adiponectin in our pediatric sample however they did not correlate with the degree of hepatic steatosis. Given “normal” adiponectin ranges have not been established and standardized in children it is possible that our patient population of children with obesity already had low levels of adiponectin. Unfortunately we did not have a control group of children without obesity for comparison.

Ultrasound is accessible and serves as a screening tool to identify hepatic fatty infiltration. In our study we found that almost 70% of children with obesity had some degree of fatty infiltration detected by US, which is higher compared to other studies where near 50% of children with obesity had some degree of steatosis [28]. This group also compared the extent of steatosis in prepubertal and pubertal children and found that pubertal children had higher scores in fatty infiltration by US. The limitation of using US in obese children lies in the technical difficulties and lack of accurate measures due to patient’s body habitus and subjectivity of the technique. While ultrasound is a good screening tool, there remains the possibility of false positive or false negative results until a more precise gradation is established. Other imaging modalities that have been used include computed tomography (CT) and magnetic resonance (MR).While MR has proven the most accurate test to assess liver fatty infiltration, this test is costly and not accessible in many institutions. The use of CT, which is more accurate when diagnosing fatty liver compared to US [29] is not commonly used because of the radiation exposure. Furthermore, using spectroscopy to quantify the amount of fat and water in the liver is more precise than conventional MR images [30–32]. Transient elastography is a non-invasive test that assesses the degree of fibrosis in chronic liver diseases including NAFL [33, 34]. Despite the accuracy of such tests, cost and accessibility remain limiting factors for general practitioners outside of tertiary care facilities.

Liver biopsies are rarely done in pediatric patients with obesity due to body habitus and procedure associated risks; however liver histology is considered the “gold standard” to establish the diagnosis of NAFL and we recognize this as a limitation in our study. A potential bias in our study was the inclusion of patients on medications that could affect metabolism of lipids and glucose; we elected to perform the analyses as the number of such patients was very small and findings on these patients did not differ from the entire cohort. Another limitation was the inability to pursue multiple logistic regression analyses due to the high prevalence of NAFL in our sample. As such, we cannot make conclusions about unconfounded associations in our study.

## Conclusion

There is a high prevalence of NAFL in children and youth with obesity based on US findings and abnormal ALT. Additionally, elevated serum TG was an associated risk factor for NAFL. These findings may help clinicians identify high risk patients. Further efforts for effective interventions are required on this patient population as they are at a high risk of progressive liver disease in adulthood.

## Abbreviations

NAFL: Non-Alcoholic Fatty Liver
BMI: body mass index
ALT: alanine aminotransferase
AST: aspartate aminotransferase
HOMA: Homeostasis Model Assessment
LDL-C: low-density cholesterol
TG: triglycerides
HDL-C: high-density cholesterol
CT: computed tomography
MR: magnetic resonance
